# Improving the Thermal Behavior and Flame-Retardant Properties of Poly(*o*-anisidine)/MMT Nanocomposites Incorporated with Poly(*o*-anisidine) and Clay Nanofiller

**DOI:** 10.3390/molecules27175477

**Published:** 2022-08-26

**Authors:** Mirza Nadeem Ahmad, Sohail Nadeem, Mohsin Javed, Shahid Iqbal, Sadaf ul Hassan, Samar O. Aljazzar, Eslam B. Elkaeed, Rami Adel Pashameah, Eman Alzahrani, Abd-ElAziem Farouk, Mohammed T. Alotaibi, Hisham S. M. Abd-Rabboh

**Affiliations:** 1 Department of Applied Chemistry, Government College University Faisalabad, Faisalabad 38000, Pakistan; 2 Department of Chemistry, School of Science, University of Management and Technology, Lahore 54770, Pakistan; 3 Department of Chemistry, School of Natural Sciences (SNS), National University of Science and Technology (NUST), H-12, Islamabad 46000, Pakistan; 4 Department of Chemistry, College of Science, Princess Nourah bint Abdulrahman University, P.O. Box 84428, Riyadh 11671, Saudi Arabia; 5 Department of Pharmaceutical Sciences, College of Pharmacy, AlMaarefa University, Riyadh 13713, Saudi Arabia; 6 Department of Chemistry, Faculty of Applied Science, Umm Al-Qura University, Makkah 24230, Saudi Arabia; 7 Department of Chemistry, College of Science, Taif University, P.O. Box 11099, Taif 21944, Saudi Arabia; 8 Department of Biotechnology College of Science, Taif University, P.O. Box 11099, Taif 21944, Saudi Arabia; 9 Department of Chemistry, Turabah University College, Taif University, P.O. Box 11099, Taif 21944, Saudi Arabia; 10 Chemistry Department, Faculty of Science, King Khalid University, P. O Box 9004, Abha 61413, Saudi Arabia; 11 Department of Chemistry, Faculty of Science, Ain Shams University, Abbassia, Cairo 11566, Egypt

**Keywords:** MMT (montmorillonite) clay, (poly)*ortho*-anisidine, nanocomposites, nanofiller, HBT

## Abstract

The synthesis of MMT and poly(*o*-anisidine) (MMT/POA) clay nanocomposites was carried out by using the chemical oxidative polymerization of POA and MMT clay with POA, respectively. By maintaining the constant concentration of POA, different percentage loads of MMT clay were used to determine the effect of MMT clay on the properties of POA. The interaction between POA and MMT clay was investigated by FTIR spectroscopy, and, to reveal the complete compactness and homogeneous distribution of MMT clay in POA, were assessed by using scanning-electron-microscope (SEM) analysis. The UV–visible spectrum was studied for the optical and absorbance properties of MMT/POA ceramic nanocomposites. Furthermore, the horizontal burning test (HBT) demonstrated that clay nanofillers inhibit POA combustion.

## 1. Introduction

The area of science and competition in the preparation of better materials with important characteristics is steadily expanding, and particularly in nanotechnology areas, a vast quantity of studies and improvement has been observed over the last few centuries [1,2,3]. Nanotechnology is becoming the largest area of growth in the world [4]. This technology can be used in all areas of science, and so nanotechnology is a multidisciplinary technology [5,6,7]. Nanotechnology effectively addresses products with at least one aspect in the nanometer spectrum [8,9,10,11]. Nanotechnology involves buildings and systems with fresh characteristics owing to the configuration of their atoms [12,13,14]. Nanocomposites are, in fact, mixtures in which one of the parts at the nanometer stage is available. Differences in particle characteristics can be seen when the particle size is below a certain point; this point is the nanometer stage. This is essential for enhancing characteristics, and these characteristics can be used for product enhancement [15,16,17].

In order to immediately recognize performance upgrades (thermochemical, limited, fireproof capability) with the filler-dispersion levels and the quality of the relations of the nanoparticle’s solution, the nanocomposites have an obvious effect on their macroscopic characteristics. These nanocomposites display fresh component characteristics and are likely to become prospective applicants in different areas, such as corrosion safety, catalysis, electrode equipment, biosensors, storage equipment, and sensors [18,19,20]. In the past century, the strength, rigidity, and high aspect ratio of nanocomposites were high. Nanocomposites have been examined through chemical polymerization and humidity detecting features. Polymer clay nanocomposites have been found to enhance the gas obstacle, thermal stability, mechanical resistance, and flame retardancy [21,22,23].

Poly(*ortho*-anisidine) POAS/MWCNT compares this with plain Polymer POAS conduction to verify the actual progression in the polymer matrix owing to the existence of MWNT [24,25,26]. MMT clay is one of the smectite clusters, with a framework made of an octahedral sheet, which is sandwiched between tetrahedral silicate sheets [26,27,28]. It is the most commonly used as characteristic dirt and has been efficiently interlinked in different nanocomposite frameworks [29]. It has been used as both an excipient and a vibrant ingredient in pharmaceutical products. In comparison with Pt backed up with POA/MWCNT for formaldehyde oxidation, poly(*o*-anisidine)/MWNT was proven to have stronger catalytic activities [30]. To decrease the very costly Pd price and enhance their catalytic performance, it should be highlighted that Pd-based bimetallic nanostructures are particularly important as low-cost anode catalysts due to their reduced prices, increased density, and increased resistance to poisoning.

In this work, we show that the addition of mono (*o*-anisidine) (POAS) to CNTs can make excellent HCl molecular transmitters feasible. Following exposure to small HCl concentrations in the environment, the POAS-modified CNT test shows significant electric-conductance modulation. Especially at room temperature, these advanced sensor characteristics are accomplished. Furthermore, we demonstrate that CNT and nanocomposites of poly(*o*-anisidine) can be easily scaled up through a simple growth approach with a collection of CNTs for self-assemblies. The existence of polymeric-matrix CNTs appears to lower the unpoetic form’s insulating capacity, but they cannot produce a large increase in the conductive features [31]. For doped POA and POA/CNT, the specific conductivity of pure polymers for all samples of different thicknesses is nearly the same magnitude. CNTs have insufficient capacitance characteristics.

## 2. Experimental

### 2.1. Materials and Chemicals

*o*-anisidine, double-distilled water, ammonium peroxy disulfate (APS), ethanol (CH_3_CH_2_OH) HCl, diaminodiphenylamine (DDPA), ammonium persulfate ((NH_4_)_2_S_2_O_8_), and montmorillonite clay (MMT clay) were used. All of the chemicals were bought in analytical grades and put to use right away.

### 2.2. Preparation of Poly(o-anisidine)

**Figure 1:**
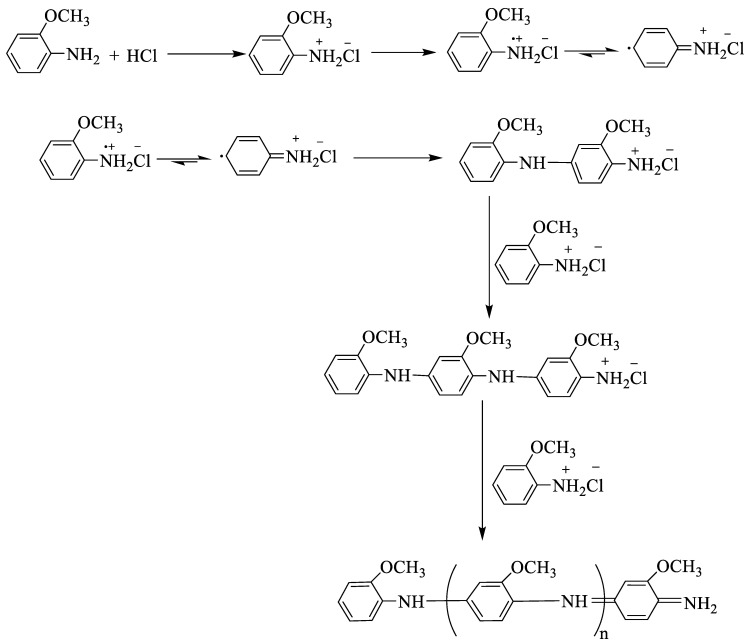
Mechanism of polymerization of *o*-anisidine.

POA was prepared by the chemical-oxidative-polymerization technique (Figure 1). An amount of 0.2463 g of POA in 20 mL of 1 M HCl was used to prepare Solution A. An amount of 0.02463 g of DDPA was added to Solution A. An amount of 0.4563 g of APS in 20 mL of 1 M HCl was used to produce Solution B. Solution A was kept in an ice bath by magnetic agitation. Subsequently, Solution B was inserted dropwise to Solution A on continuous stirring for 3 h. The color of the solution turned green, and then the agitation was stopped. Then, it remained undisturbed for some time. The product was purified with 1 M of HCl, and then with distilled water. The product was added to a Petri dish and was kept in the oven overnight at 80 ℃. The sample was obtained as a powdered dry form.

### 2.3. Preparation of MMT/POA Clay Nanocomposite

Nanocomposites of MMT/POA clay were constructed by the chemical-oxidative-polymerization technique, as mentioned for the POA. Different desired percentages of MMT concerning POA clay were added at the time of polymerization (i.e., 2%, 4%, 6%, 8%, and 12%). The percentage of MMT clay was taken by keeping in view the weight of the *o*-anisidine monomer, as mentioned in Table 1. The *o*-anisidine monomer was dissolved in a 1 M HCl solution, and DDPA was added afterward. The mixture was mixed with MMT clay and was sonicated for 15 min for uniform dispersion. The solution was continuously stirred for complete mixing. Another solution of APS was prepared by adding it to 20 mL of 1 M HCl. This solution acted as an oxidant and was added dropwise to the above mixture. Afterward, the mixture was stirred for 3 h for complete mixing. The reaction temperature was maintained at 0–4 °C. Keeping in mind the above process, all the composites of the desired percentages of MMT clay were prepared. Finally, the samples were centrifuged and washed using distilled water, and then with ethanol. Then, the samples were dried in an electric oven at 80–100 °C, and they were obtained in dry-powder form. Consequently, the characterization was performed by using FTIR, SEM, and UV–visible spectroscopy.

## 3. Result and Discussion

### 3.1. FTIR Spectroscopy Studies

**Figure 2:**
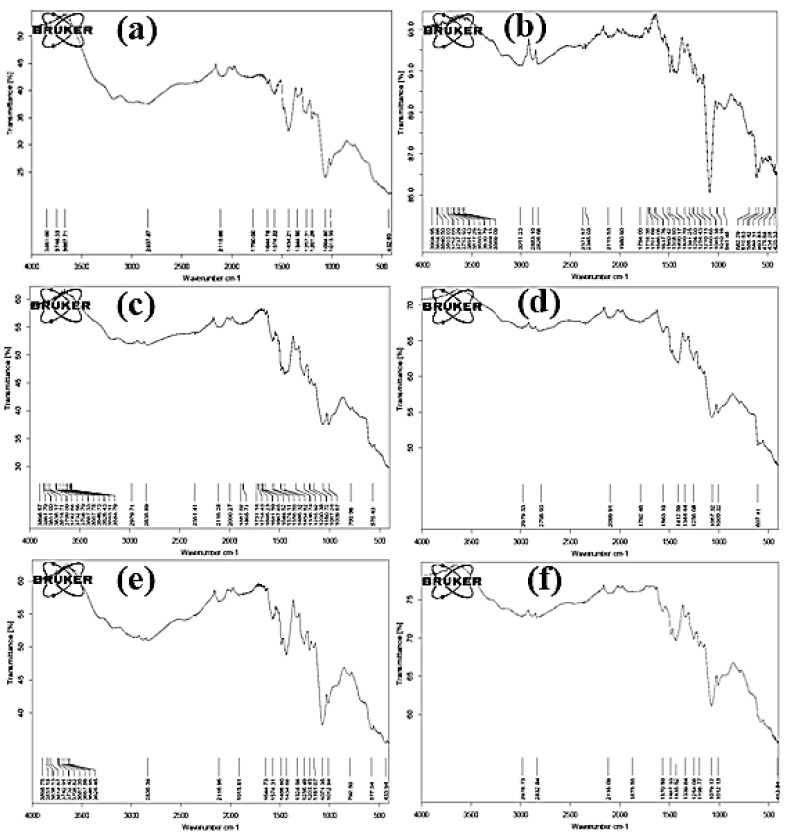
FTIR spectra of: (**a**) POA; (**b**) 2% POA/MMT; (**c**) 4% POA/MMT; (**d**) 6% POA/MMT; (**e**) 8% POA/MMT; (**f**) 12% POA/MMT.

The FTIR analysis of the MMT/poly(*o*-anisidine) clay nanocomposite was obtained by using an Agilent 630 carry spectrometer. The FTIR values of pure polymers and nanocomposites were recorded, and the peaks of the values are shown in the graph [32]. Fourier transform infrared spectroscopy is an apparatus and strategy that helps to estimate and understand what type of groups and elements are attached to samples [33]. Figure 2a shows the FTIR spectra of pure POA. The strong peaks appeared at 3467cm^−1^, which showed the presence of N-H. The peaks at 1644 and 1574 cm^−1^ indicated that the POA contained the spine’s benzene ring, and these clusters also indicated that the poly(*o*-anisidine) was neutralized at the counter ion load. The C-N elongation method correlated the 1345 cm^−1^ and 1255 cm^−1^ spikes. The spectra (Figure 2b) show the presences of MMT glue with 2% MMT resin, 1067 cm^−1^ elevated ribbon, and 1002 cm^−1^ bands [34].

In Figure 2c, the FTIR spectra suggest that the peak range started at 1644 and 1574 cm^−1^, and they show that the polymers with the aid of 4% MMT material were nearly the same as 2%, showing that 4% of the MMT glue was used to reinforce the plastic crystallinity. The active started with O-H 3011 cm^−1^, and CH_2_ bands at 2826 cm^−1^ and 2371 cm^−1^. In Figure 2d, the spectra show that the acid surface with 1067 cm^−1^ and 1009 cm^−1^ was distinguished by waves of MMT clay by adding six times MMT clay in the polymer matrix. The bottom frequency part is assigned a maximum of 2979 cm^−1^ and 2735 cm^−1^ for the elongation waves of N-H.

In Figure 2e, the spectra indicated the increase in C-O-C, OR-C-C, bonded C-H to CH_2_, and the C=O elongation was due, with 8% of the MMT clay that was added to a polymer, and the maximum peaks were discovered at 1071 cm^−1^, 1163 cm^−1^, 1430 cm^−1^, and 1625 cm^−1^. Bands of 2836 cm^−1^ were assigned to the asymptomatic extended C-H of CH_2_ and CH_3_ of MMT/POA. In Figure 2f, the spectra show the 1644 and 1574 cm^−1^ bands that showed the presence of benzenoids and quinoids in the form of polymers, to which were added 12% MMT clay to the polymeric matrix. These bands indicated that the counter-ion charges were neutralized in the poly(*o*-anisidine) chain. Based on the present moisture, the highest range was 3667 cm^−1^, which showed the presence of NH-.

### 3.2. UV–Visible Absorption of POA and MMT/POA Clay Nanocomposites

**Figure 3:**
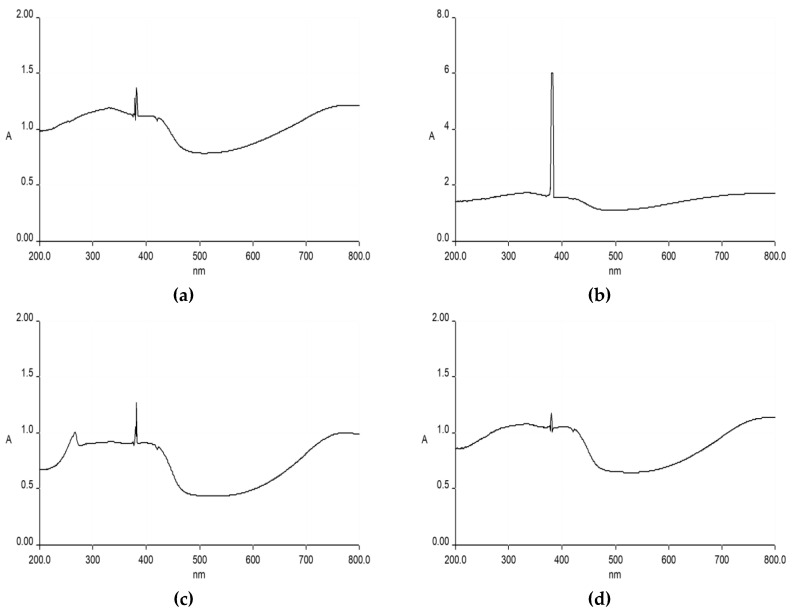

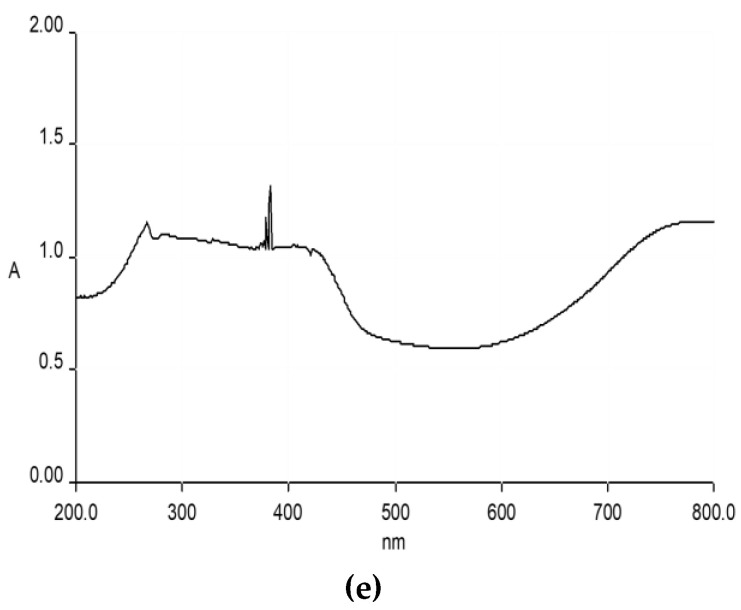
UV–vis: (**a**) 2% MMT/POA; (**b**) 4% MMT/POA; (**c**) 6% MMT/POA; (**d**) 8% MMT/POA; (**e**) 12% MMT/POA.

To determine the absorption characteristics of the MMT/POA clay composites, UV–visible spectroscopy was used. [35]. The spectrophotometer was provided with UV Win Lab programming for the recording and analysis of the data. The fundamental spectrophotometer correction was conducted using an empty reference. Some early researchers in UV–visible spectral studies have measured the interaction and quantities of poly(*o*-anisidine)/MMT pottery. In Figure 3a, the poly(*o*-anisidine) intercalated in the toner pits indicated the UV–visible variety with the aid of 2% MMT of clay. At 340 nm, a strong peak was found for the UV absorption, and the normal change n fi p* is provided for poly(*o*-anisidine). In Figure 3b, after the mechanical reaction of poly(*o*-anisidine)/MMT with the stoichiometric oxidizing force ((NH_4_)_2_S_2_O_8_), the poly(*o*-anisidine) completely disappeared in the 4% MMT composite. There were two new poly(*o*-anisidine) values, one at 420 nm, and the other at 800 nm, for the HCl-doped poly(*o*-anisidine).

In Figure 3c, the MMT clay with the poly(*o*-anisidine) nanocomposite existed with an excess of 6% of MMT clay [36]. The polymerization of polymer and MMT clay was completely homogenized. The maximum peak was at 351 nm, and the atomic changes were linked with the benzene and quinoid clusters. However, at 439 nm, they were associated with small and high-power polar caps. In Figure 3, the spectra 3d and 3e show two new severe highs assigned to p-p*, and for the others, 650 nm due to p-polar transformation with the aid of 8 and 12% MMT sand, respectively. The UV–visible spectral study assessed the amount of poly (*o*-anisidine) charged in the clay.

### 3.3. Scanning-Electron-Microscopy (SEM) Analysis

**Figure 4:**
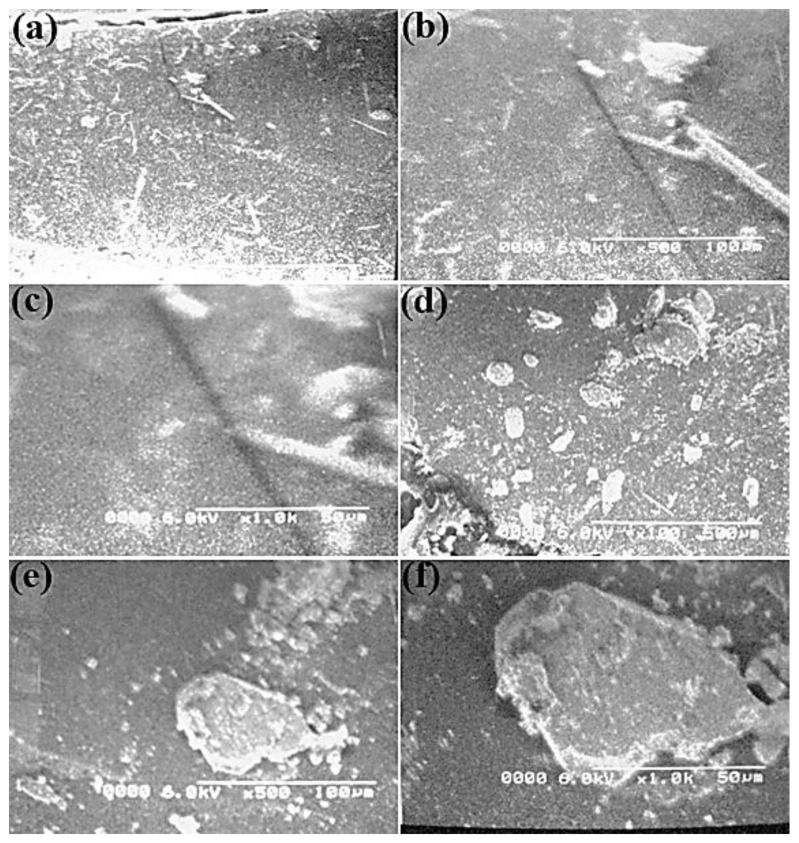
SEM images at different magnifications of POA (**a**–**c**), and POA with 2% MMT clay (**d**–**f**).

SEM evaluation represents a powerful tool for researchers that uses a focused electron beam to produce complex and high-large images of the topography sample layer [37,38]. A mechanochemical intercalation method with different POA components, together with intact MMT clay, was used to register the SEM micrographs of the poly(*o*-anisidine)/MMT clay nanocomposites. In Figure 4a–c, the SEM images show the poly(*o*-anisidine) ground morphology. A crushed rock indicated the sheer POA. In Figure 4d–f, the semi-micrographs of the NCF film are displayed at separate magnifications by the addition of 2 % MMT clay. The growth of polymer clay nanocomposites was ended with an SEM micrograph. Because of the insufficient MMT clay, the existing POA ground was not intercalated. This shows that the polymer was in lighter regions.

**Figure 5:**
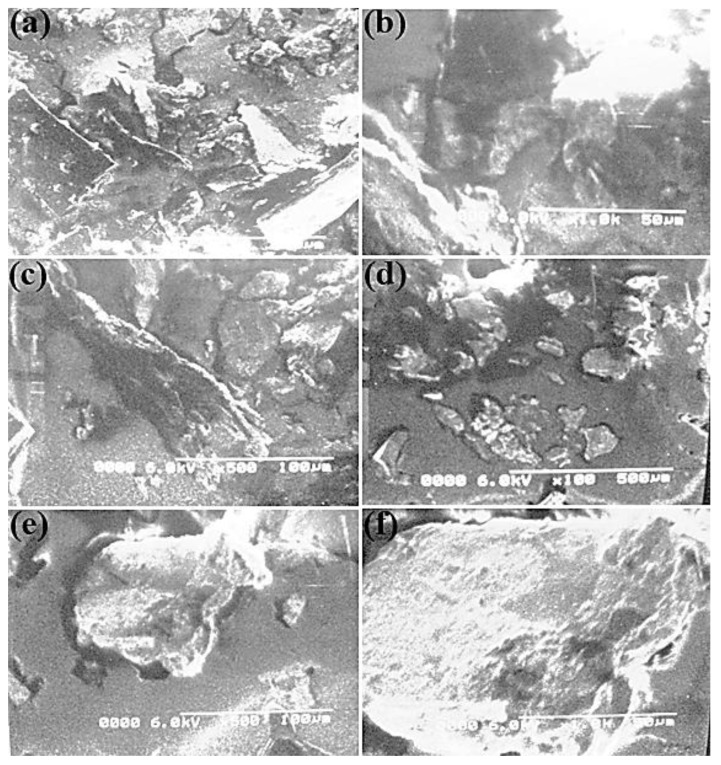
SEM images at different magnifications of POA with 4% MMT clay (**a**–**c**), and POA with 12% MMT clay (**d**–**f**).

Figure 5a–c shows the semi-microwave properties of the ceramic strands and the light regions of the fabric indicated by the black constructions of 6% of MMT material. In the different paths, the irregular allocation of the strands of tone was observed. The polymer matrix indicated that the organized composition was distressing, and the clay parts dispersed. The disorderly structure of the clay strata was better at increased magnification. In Figure 5a–c, some lighter regions in the micrographs, the existence of some intercalated parts or feasible edge-to-edge relationships of some ceramic phases were still discovered in comparison with 6% of MMT material. These interactions may include emulsion-based nanocomposites, including particularly hydrophilic clays.

In Figure 5d–f, the structure of POA was noticed by adding roughly 50–100 nm of nanofibers (i.e., by adding 12 parts of MMT gum). The clusters of POA contained sheets of clay with caught POA fibers. The image presents a standardized nanocomposite layer of POA clay due to the intercalation of POA and wood. In Figure 5d–f, the combination of POA with clay in nanocomposites is demonstrated by a 12-fold quantity of MMT clay. However, in a nanocomposite film, the dispersion of clay was not consistent. In comparison with the POA clay, strong interface binding between the POA and sand revealed higher mechanical and heat features. Compared with natural POA, the POA surface area of nanocomposites was enhanced. This is owing to the inclusion of nanofillers that causes the polymer matrix and fillers to interact more closely.

### 3.4. TGA/DSC Evaluation of Pure POA and MMT/POA Clay Nanocomposites

The thermal stability of the investigated polymers was assessed using TGA/DSC. The thermal properties of pure POA and its nanocomposites (MMT/POA) were examined using thermogravimetric analysis. TGA (thermogravimetric analysis) is often used to examine weight loss as a function of time, and especially temperature, in order to study the degradation of polymeric materials. The thermal stability may be improved by adding nanofiller clay to the polymer matrix. It is extremely elegant and crucial for commercial reasons because the products can be prepared for less money, with a simpler process, and with less weight, even though it represents the possibility of achieving a significant increase in thermal stability by using only a small amount of MMT clay in the finished product [25,39,40]. According to the DSC/TGA measurements, the inclusion of dispersed MMT clay in the polymer matrix considerably improved the flame retardancy and thermal stability of MMT/POA clay nanocomposites compared with pure POA [41].

**Figure 6:**
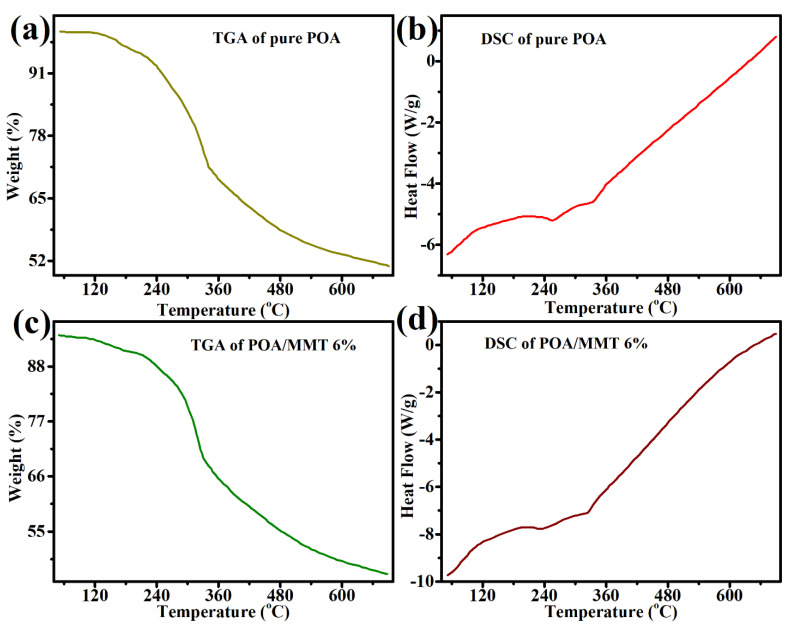
TGA/DSC of pure POA (**a**,**b**), and MMT/POA at 6% (**c**,**d**).

The elimination of H_2_O molecules causes the initial weight loss at 50 °C, and continues up to 110 °C, according to the thermogram of pure POA and MMT/POA nanocomposites. The loss of HCl was detected at 200 °C, followed by fast mass loss until 620 °C [42]. POA breakdown is continuous up to 700 °C (Figure 6). POA nanocomposites have superior heat stability compared with pure polymers, according to this thermogravimetric research (poly(*o*-anisidine)). The inserted polymer’s consistency and strength are related not only to its unique structure, but also to the steric considerations that prevent the portions sandwiched between MMT clay layers from thermally moving [43]. The superiority of the benzenoid structure explains the higher thermal stability of nanocomposites. Pure POA, in contrast, has a quinoid ring in its structure, which contributes to its poor thermal stability. POA decomposes continuously up to 700 °C; however, the total degradation of MMT/POA clay nanocomposites does not occur until beyond 700 °C [42]. When POA and MMT/POA nanocomposites decompose, some char is produced. This means that the presence of -Cl has prevented the whole breakdown from taking place. Lower weight loss was shown by comparing POA and POA/MMT clay nanocomposites with varying mass weights. POA has 50% residue (Figure 6a,b), while POA/MMT clay nanocomposites with 6% have 58% residue (Figure 6c,d). The thermal stability of POA was improved using MMT clay nanofiller in this study. POA polymer decomposes as the temperature is elevated; however, adding MMT clay to the polymer does not dissolve it due to the MMT nanofiller’s excellent thermal resilience [44]. By increasing the quantity of MMT clay, the percentage of the residual steadily rose.

### 3.5. Horizontal Burning Test

**Figure 7:**
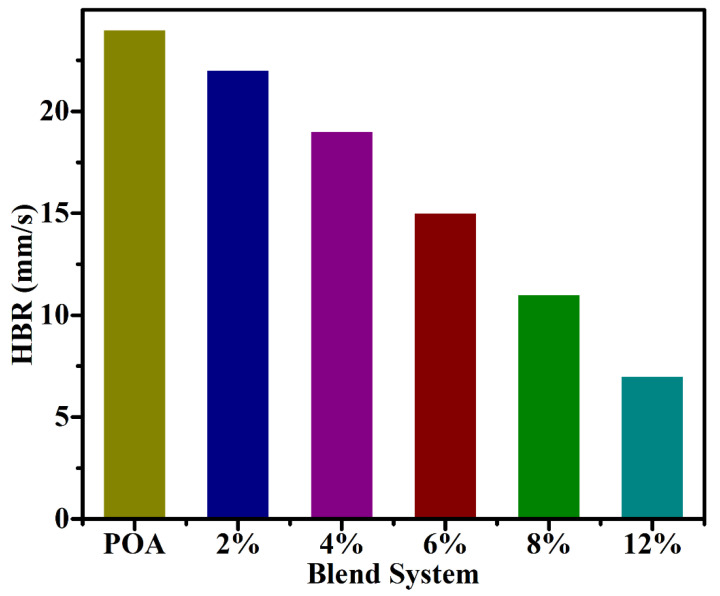
The horizontal burning rate of pure POA blends with MMT clay nanofiller (2, 4, 6, 8, and 12%).

A strip of PCO/MMT blend was created in a Teflon mold for the horizontal burning test (118 mm × 7 mm × 1 mm). The strip was hung horizontally from the stand. For each blend system, the opposite end of the strip was lit with a burner, and the time it took to burn from Mark A to B was recorded [45,46,47]. The blend of MMT/POA at 12% has a 3.4-fold lower burning rate than pure POA (Table 2). The clay nanofillers’ ability to delay the POA heat deterioration was validated by this finding. The trend is linear, which means that, as the fraction of additive increases, the rate of burning decreases, and vice versa (Figure 7).

### 3.6. Cone-calorimeter Test

Pure POA and MMT have been manufactured and evaluated in the cone calorimeter with the aim of evaluating the fire efficiency of the MMT/POA in terms of the kind and integration of the nanofillers. (2, 4, 6, 8, and 12 mass percent loading) [48,49,50]. By altering the chemistry in the condensed phase and physical burning process, inorganic flame-retardant additives such as MMT usually increase the flame retardancy of polymeric materials [51]. As a result, it is predicted that adding nanoclay to the POA matrix will improve the MMT/POA’s flame-retardancy performance when compared with pure POA in the cone-calorimeter test, and the results would be comparable to earlier smaller-scale thermal investigations [52]. For a well-established fire scenario, a cone calorimeter offers a comprehensive portrayal of the fire-response qualities. Data from cone calorimeters may be employed to equivalence the fire behavior of various materials, provide data for modeling the behavior of fires in the real world, and also to establish the parameters needed for regulatory reasons, such as the total heat evolved, heat-release rate, and fire-growth-rate index (FIGRA) [53]. The heat-release rate (HRR) is considered to be the utmost important of these fire-response qualities because it is the factor that drives the spread of fire. The total heat release, effective heat of combustion, duration to ignition, mass loss rate, smoke-specific extinction or obscuration area, and CO and CO_2_ production are other cone-calorimeter characteristics that are examined. Table 3 lists the findings from the cone calorimeter for pure POA and MMT/POA nanocomposites.

## 4. Conclusions

With the oxidative-polymerization technology and the coarse protective components of their POA mixture with MT sand, MMT/POA resin nanocomposites were efficiently generated with 2%, 4%, 8%, and 12%. This study demonstrated that the POA layer has excellent protective properties for rust and can be considered as a future-oriented covering fabric to protect noncorrosion materials in aqueous environments. The cumulative and electronic steric effects of the important methoxide substitute were attributable to the reduced conductivity at room temperature of the POA. Enhanced product conductivity can be used with the benefit of elevated solubility in predominantly alcoholic solvents for several technical purposes. FTIR and SEM described the nanocomposite composition of MMT/POA clay and its morphology, which verified the MMT/POA-nanocomposite growth. The UV–visible spectrum has been studied for the optical and absorption qualities of MMT/POA ceramic nanocomposites.

## Figures and Tables

**Table 1:** Scheme of nanocomposite preparation.

Sr. No.	Monomer Poly(*o*-anisidine)	APS (Ammonium Persulphate)	DDPA	MMT Clay	% of Filler in Composite
1	0.0255 g	0.456 g	0.002555 g	0 g	0%
2	0.0255 g	0.456 g	0.002555 g	0.00051 g	2%
3	0.0255 g	0.456 g	0.002555 g	0.000102 g	4%
4	0.0255 g	0.456 g	0.002555 g	0.00153 g	6%
5	0.0255 g	0.456 g	0.002555 g	0.00204 g	8%
6	0.0255 g	0.456 g	0.002555 g	0.00306 g	12%

**Table 2:** The horizontal burning rates for MMT/POA nanocomposite.

Nanocomposite	POA	2%	4%	6%	8%	12%
Burning Time/73 mm	16	25	33	47	58	75
HBR (mm/s)	24	22	19	15	11	7

**Table 3:** Cone-calorimeter tests on the heat and ignition characteristics of pure POA and MMT/POA nanocomposites.

Samples	Effective Heat of Combustion, MJ kg^−1^	Time to Ignition, t_ing_/s	Flame-Out Time, s	Mean CO_2_ Yield, kg kg^−1^	Average Mass Loss Rate (MLR), avg/g s^−1^ m^−2^	Total Heat Release (THR), MJ m^−2^
POA	27.4	11	486	2.3	25.4	131.5
2% MMT/POA	29.6	10	541	2.3	29.5	134.6
4% MMT/POA	31.8	17	606	2.4	35.6	137.4
6% MMT/POA	34.2	24	649	2.4	39.8	140.7
8% MMT/POA	33.5	19	681	2.3	28.4	139.4
12% MMT/POA	32.7	13	723	2.2	17.6	136.7

## Data Availability

The data will be available on request.
